# Polymorphisms in the HTR2A and HTR3A Genes Contribute to Pain in TMD Myalgia

**DOI:** 10.3389/froh.2021.647924

**Published:** 2021-03-02

**Authors:** Sofia Louca Jounger, Nikolaos Christidis, Britt Hedenberg-Magnusson, Thomas List, Peter Svensson, Martin Schalling, Malin Ernberg

**Affiliations:** ^1^Division of Oral Diagnostics and Rehabilitation, Department of Dental Medicine, Karolinska Institutet, Huddinge, Sweden; ^2^Scandinavian Center for Orofacial Neurosciences (SCON), Huddinge, Sweden; ^3^Department of Orofacial Pain and Jaw Function, Folktandvården Eastmaninstitutet, Stockholm, Sweden; ^4^Department of Orofacial Pain and Jaw Function, Faculty of Odontology, Malmö University, Malmö, Sweden; ^5^Section of Orofacial Pain and Jaw Function, Department of Dentistry and Oral Health, University of Aarhus, Aarhus, Denmark; ^6^Center for Molecular Medicine, Karolinska Institutet, Stockholm, Sweden

**Keywords:** serotonergic genes, polymorphisms, myalgia, temporomandibular disorders, psychological distress, pain

## Abstract

**Background:** The aim of this study was to investigate if single nucleotide polymorphisms (SNPs) related to monoaminergic neurotransmission, in particular the serotonergic pathway, contribute to pain perception in patients with temporomandibular disorder (TMD) myalgia and if there is a correlation to jaw function as well as psychosocial factors such as stress, anxiety and depression.

**Materials and Methods:** One hundred and seventeen individuals with TMD myalgia were included. A venous blood or saliva sample was taken for genetic analyses and genotyped regarding *HTR2A (rs9316233) HTR3A (rs1062613), HTR3B (rs1176744), SERT (5-HTTLPR)* and *COMT (rs4680)*. A clinical examination according to Diagnostic Criteria for TMD (DC/TMD) was performed and axis II data (psychosocial factors) were compared between participants with different genotypes for each gene using Kruskall–Wallis test. The characteristic pain intensity (CPI) was tested for correlations to scores for the Perceived Stress Scale, Generalized Anxiety Disorder, and Patient Health Questionnaires using Spearman's rank correlation test with Bonferroni correction for multiple testing. To further explore data factor analysis was performed to identify latent factors associated to the outcome variables.

**Results:** Participants carrying at least one copy of the rare allele of the *HTR2A (rs9316233)* and *HTR3A (rs1062613)* had higher CPI compared with the participants with the homozygous common genotype (*P* = 0.042 and *P* = 0.024, respectively). Correlation analyses showed several significant positive correlations between CPI on one hand, and self-reported psychosocial distress and jaw function on the other hand for several genotypes that mostly were weak to moderate. The factor analysis identified two latent variables. One was positively correlated to the *HTR3B* gene, jaw function and self-reported parafunctions, and the other was positively correlated to psychological distress and negatively correlated to *SERT*.

**Conclusion:** Taken together, the polymorphism *rs1062613* in the *HTR3A* gene contributes to pain intensity in TMD myalgia. This together with positive interactions between pain variables and psychological factors in genotypes strengthens that pain and psychological distress are related. Further research is needed to explore this as well as the influence of gene-to-gene interactions on pain and psychological distress.

## Introduction

Chronic musculoskeletal pain conditions are a major public health problem affecting nearly one-third of the world's population [1]. Modern pain research has attracted attention to the role of the central nervous system, its neurotransmitters and genetic variations in chronic pain [2]. The large inter-individual variation in pain perception, drug response and the risk of developing chronic pain conditions are thought to be explained, at least in part, by genetic factors and how they interact within the central nervous system [2].

Temporomandibular disorders (TMD) are a type of chronic pain conditions that affects around 10–15% of the adult population. The most common subgroup is TMD myalgia with a prevalence of 5–10% [3] and is more common in women [4]. Common signs and symptoms are restricted mouth opening, pain upon chewing, pain referral, and headache, which reduces patients' quality of life [5]. It is associated with significant individual suffering, as well as annual costs to society in terms of lost work days and sick leave [6]. The pathophysiological mechanisms that underlie TMD myalgia and why it is more prevalent in women are still not understood. However, several risk factors are thought to be involved such as psychosocial, autonomic, and genetic factors [7–9].

Several genes and single nucleotide polymorphisms (SNPs) that influence pain perception have been identified [7] and at least 358 genes are thought to be relevant in pain and hyperalgesia [10]. In TMD pain, the role of genetic factors has been discussed as an important part of the etiology since several polymorphisms have shown to be associated with a higher or lower risk of TMD [8]. For example, previous studies show associations between TMD and SNPs in the *HTR2A (rs9316233)* and *COMT* genes *(rs174697)* [10–12]. Further, in another study the C allele (common genotype) in the *HTR2A* SNP *(rs2770304)* was associated with increased risk for sleep bruxism suggesting that serotonin (5-HT) and other neurotransmitters in the central nervous system and their related genes could be involved in the pathogenesis of bruxism [13]. Another twin-study revealed that TMD pain and neck pain can in part be attributed to genes [14]. This is of interest since research have shown that self-reports of oral behaviors, such as tooth-clenching and/or grinding are risk factors to TMD [15] although bruxism as such is not directly associated with TMD pain [16]. It has been hypothesized that repeated muscle contraction may cause the release of analgesic and inflammatory biomarkers that trigger nociceptors and thus may initiate and perturb TMD myalgia [17]; however, repeated muscle contractions may also lead to training effects and a decrease in self-reported muscle symptoms [18]. Irrespective of the source it is interesting to consider that elevated muscle levels of 5-HT, glutamate and cytokines have been reported in TMD myalgia [19–21].

5-HT as well as dopamine are important neurotransmitters in the central nervous system with various functions including regulation of mood, appetite, sleep, cognitive functions but are also involved in pain transmission, pain perception, and pain inhibition [22, 23]. Studies suggest that genetic variations in the serotonergic and dopaminergic systems contribute to pain sensitivity and treatment response [10, 24]. Other studies show that blocking the 5-HT_3_ receptor reduces experimental and clinical pain, but with a large inter-individual variation in the efficacy, which might be due to genetic factors [25].

A previous study supports the role of polymorphisms in the *HTR2A* gene in the genetic predisposition to musculoskeletal pain [26]. Other studies demonstrate that *HTR2A* polymorphisms show an association to major depression disorders [27] and increased psychological symptoms of dementia in patients with Alzheimer's disease [28]. Furthermore, *HTR3A/B* polymorphisms have been associated to psychiatric disorders, and a few studies have shown an association to chronic pain [29]. For example, a polymorphism in the *HTR3B* gene *(rs1176744)* was correlated to major depression in Japanese women and bipolar disorder [30]. This is a common variant found in approximately 40% of the northwest European population [30]. Since pain and depression to some extent have overlapping neural pathways, there are reasons to believe that the 5-HT-polymorphisms also may be involved in the pathophysiology of chronic myalgia [31]. Indeed, a polymorphism *(rs1176744)* in the *HTR3B* gene was associated with pain catastrophizing, supporting a role of 5-HT pathways in pain conditions [32].

The serotonin transporter (5-HTT; *SERT*) is a key regulator of serotonin metabolism. *SERT* inactivates serotonin by reuptake from the synaptic cleft and has been identified on pre-synaptic nerve terminals. Several polymorphisms in the promoter region of the *SERT* gene that affects the expression of *SERT* have been identified [33]. The *5-HTTLPR* polymorphism for example in the promoter region of the SLC6A4 gene, encodes the serotonin transporter and consists of a short (S) and a long (L) variant [34]. This SNP (*5-HTTLPR)* has been associated to anxiety disorders [35], chronic pain conditions such as fibromyalgia [36], irritable bowel syndrome [37], and tension type headaches [38].

The *COMT* gene codes for the *COMT* enzyme, which breaks down certain neurotransmitters such as dopamine in the brain's prefrontal cortex. A common polymorphism is the *Val158Met (rs4680)* were the common allele is guanine (G), coding for a valine amino acid. In this polymorphism G is substituted by alanine (A) which changes the amino acid to a methionine. As a result, the A allele carriers have more dopamine in their prefrontal cortex, which may be responsible for many of the neuropsychological associations. For example, a recent study suggests that this polymorphism may influence cognitive vulnerability to depression [39]. The same polymorphisms have also been shown to play a key role in pain sensitivity in fibromyalgia and patients with chronic widespread pain [40]. Furthermore, in patients with Parkinson's disease, carriers of the homozygous common (G/G) and heterozygous (A/G) genotypes had significantly higher pain severity than patients with the homozygous rare (A/A genotype) suggesting that the *COMT rs4680* contributes to both pain susceptibility and severity in patients with Parkinson's disease [41].

With this in mind, the aim of this study was to investigate if polymorphisms in the *HTR2A (rs9316233), HTR3A (rs1062613), HTR3B* (*rs1176744), SERT (5-HTTLPR)* and *COMT (rs4680)* genes contribute to pain perception in TMD myalgia and if pain and psychosocial factors such as stress, anxiety and depression correlate. A second aim was to explore possible interactions between gene variants and outcome measures. We hypothesized that genotypes in these polymorphisms contributes to higher pain characteristics in TMD myalgia patients suggesting the importance for pain transmission and finally that pain and psychosocial factors correlate.

## Materials and Methods

The project followed the guidelines according to the Declaration of Helsinki as well as Good Clinical Practice and was approved by the Regional Ethical Review Board in Stockholm, Sweden (2011/1955-31/2) and by the Swedish Data Protection Authority (Dnr 54-2013). All participants received written and verbal information of the study before inclusion and gave their written consent.

### Participants

One hundred and nineteen individuals with TMD myalgia were consecutively included in this study. Patients with TMD that were referred to the specialist clinics for Orofacial Pain and Jaw Function at the Eastman Institute, Folktandvården AB, Stockholm or the Department of Dental Medicine at the Karolinska Institutet, Huddinge, Sweden were asked about participation in the study.

An a priori power analysis revealed that 37 subjects would be sufficient to detect a group difference in the primary outcome (pain intensity) between genotypes of 1.5 SD (*t*-test) when *a* = 0.05 and *b* = 0.80 and that 30 subjects in each genotype would be sufficient to detect medium strong correlations (*r* < 0.50).

Inclusion criteria were age over 18 years, presence of facial pain > 6 months, and a primary diagnosis of TMD myalgia according to the DC/TMD criteria [42]. Since the DC/TMD allows for multiple diagnoses and this is common, patients could also have other diagnoses, e.g. other DC/TMD pain diagnoses or intra-capsular disorders. Exclusion criteria were systemic inflammatory connective tissue diseases, whiplash-associated disorder, fibromyalgia, neuropathic pain or neurological disorders, and pain of dental origin.

### Study Design

The participants were first examined according to the Diagnostic Criteria for TMD (DC/TMD) using axis I and II [42] to establish that they had a diagnosis of TMD myalgia. Axis II (psychosocial distress factors) was assessed with a standardized questionnaire that is used for all new referrals to the clinic. This included background data about demographics and medical conditions (allergies, ear-nose-throat conditions, abdominal disorders, cardiovascular disease, diabetes, migraine, mental disease (such as depression or anxiety), and several validated instruments to assess pain characteristics, psychological factors, functional status of the masticatory system and behaviors with significance for facial pain. These included the Graded Chronic Pain Scale (GCPS) the Perceived Stress Scale (PSS-10), the Generalized Anxiety Disorder (GAD-7), the Patient Health Questionnaires (PHQ-9 and PHQ-15), the Jaw Function Limitation scale (JFSL-20), and the Oral Behaviors Checklist (OBC-21). If a participant was included in the study, venous blood or saliva was sampled for genetic analyses.

#### Questionnaires

##### Graded Chronic Pain Scale

The graded chronic pain scale (GCPS) includes three subscales assessing the current as well as the average and worst pain intensity during the last month on 0–10 numeric rating scales (NRS) with the endpoints “no pain” and “maximal imaginable pain.” From the three scales, the characteristic pain intensity (CPI), i.e., the average of the three scales multiplied with 10 is calculated. The questionnaire also includes three subscales assessing pain interference in a similar manner and a question regarding the number of days with pain during the last month. Pain disability is calculated from these two latter measures combined and scored as Grade 0: none, Grade I: low intensity pain without disability, Grade II: high intensity pain without disability, Grade III: moderately limiting, and Grade IV: severely limiting [42].

##### Perceived Stress Scale

The perceived stress scale (PSS) contains 10 stress- related questions of a general nature with questions about feelings and thoughts during the last month, situations in life perceived as stressful and the current levels of stress. The scores range between 0 and 40 with a maximum scoring of 40. Higher scores indicate higher perceived stress (low stress 0–13; moderate stress 14–26; high stress 27–40) [43]. The Swedish version of the PSS-10 was used [44].

##### Generalized Anxiety Disorder (GAD-7)

The General Anxiety Disorder (GAD-7) scale is a seven-item instrument that is used to measure or assess the severity of generalized anxiety disorder. Each item asks the individual to rate the severity of his or her symptoms over the past 2 weeks. Response options include “not at all,” “several days,” “more than half the days,” and “nearly every day.” The scores range from 0 to 21. Scores of 5, 10, and 15 represent cut-points for mild, moderate, and severe anxiety, respectively [42].

##### Patient Health Questionnaires (PHQ-9 and PHQ-15)

The Patient History Questionnaires (PHQ-9 and PHQ-15) are self-administered diagnostic instruments for measuring depression and somatic symptoms. The PHQ-9 questionnaire comprises 9 questions, each scored 0–3, and the PHQ-15 contains 15 questions scored 0–2. The response options for PHQ-9 are the same as for GAD-7, whereas the options for PHQ-15 are not bothered, bothered a little, and bothered a lot. The scoring of PHQ-9 range from 0 to 27 and The PHQ-15 score ranges from 0 to 30, where the scores 5, 10, 15, and 20 represent mild, moderate, moderately severe, and severe depression, respectively. For PHQ-15 the scores 5, 10, and 15 represent low, medium, and high somatic symptoms, respectively [42].

##### Jaw Function Limitation Scale (JFLS)

The Jaw Function Limitation scale is an instrument for assessing functional status of the masticatory system (mastication, vertical jaw mobility, and emotional and verbal expression); comprising a total of 20 items. A global score of “jaw functional limitation” can be computed as the mean of the scores for items 1, 3, 6, 10, 11, 12, 13, and 19. Subscale scores for each type of functional limitation are computed, as follows: Mastication (mean of items 1–6), mobility (mean of items 7–10), verbal and non-verbal communication (mean of items 13–20) [42].

##### Oral Behaviors Checklist (OBC)

The Oral Behaviors Checklist (OBC-21) a self-report scale for identifying and quantifying the frequency of jaw overuse behaviors in the past 1 month. It comprises 21 questions, 2 questions assess oral behaviors during sleep and 19 questions measure oral behaviors during waking hours. Each question is scored from 0 to 4. For night-time behaviors the alternatives are 0 = none of the time, 1 ≤ 1 night/month, 2 = 1–3 nights/month, 3 = 1–3 nights/week, and 4 = 4–7 nights/week. For waking hours behaviors, 0 = none of the time, 1 = a little of the time, 2 = some of the time, 3 = most of the time, and score of 4 = all the time based on frequency of activity performed. The total scores range from 0 to 84 [42].

### Genotyping

Whole blood (4 mL) was collected from a peripheral vein using Vacutainer tubes containing an ethylenediaminetetracetic acid (EDTA) solution (*n* = 91). Blood was chosen as the first alternative since we also wanted to have the possibility to analyze other biomarkers in plasma. Saliva was collected using Oragene kits (OG-500, DNA Genotek Inc., Ontario, Canada) if blood could not to be drawn due to technical reasons (*n* = 26). The OG-500 kit is a reliable method for the collection, stabilization, and transportation of DNA from saliva that is comparable to blood samples for genotyping [45]. DNA was extracted from blood or saliva using standard manual methods [46–48]. In short, DNA was extracted from peripheral blood mononuclear cells and the expression of the DNA's TLRs was examined using a polymerase chain reaction. The SNPs *rs9316233 (HTR2A), rs1062613* (*HTR3A*), *rs1176744* (*HTR3B*), and *rs4680* (*COMT*) were genotyped on the Applied Biosystems Quantstudio 7 Flex Real-Time PCR System from Thermo Fischer Scientific, Carlsbad, CA by using allele specific Taqman MGB probes labeled with fluorescent dyes FAM and VIC, according to the manufacturer's protocol.

The SNP (*5-HTTLPR*) was determined with PCR reactions using Biorad Tetrade (Biorad, Hercules, CA, USA) to amplify the samples followed by an initial denaturation step for 15 min at 95°C. The primer sequence was ‘59-GGCGTTGCCGCTCTGAATGC-39’ and the reverse ‘59-GAGGGACTGAGCTGGACAACCAC-39.’ The amplification consisted of 33 cycles of 30 s denaturation at 94°C, annealing for 30 s at 63°C and elongation for 30 s at 72°C. This was followed by a final elongation for 10 min at 72°C. Another 11 μL of the PCR product was digested with 1.05 μL MSP1 (New England Biolabs, Ipswitch, MA, USA) and incubated at 37°C for 12 h. The long and short fragments were separated and visualized at 110 UV for 2 h on a 4% Agarose gel containing GelRed®. This was done according to the manufacturer's protocol in line with a previous study [49].

### Statistics

We used a stepwise approach for data analysis. Data were first analyzed with univariate statistics according to the aims using SigmaPlot for Windows, version 11 (Systat Software Inc., Chicago, IL, USA). The Shapiro–Wilk's test was used to evaluate if data were normally distributed. As most data were not normally distributed and/or ordinal, non-parametric statistics were used. Descriptive data are presented as number of participants (*n*), frequencies (%), and median with interquartile range (IQR) depending on type of data. The level of significance was set to *P* < 0.05. The Hardy–Weinberg equilibrium was evaluated for each SNP using a χ^2^-test and significant *P*-values were corrected for multiple testing (Holm-Sidak). Participants with at least one copy of the rare allele (the homozygous rare and the heterozygous) were combined in the analyses and compared to the common genotype. The primary outcome variable was CPI for the participants' genotypes. Presence of psychological distress and parafunctions were secondary outcomes. To analyze differences between genotypes in pain variables, psychological distress and jaw function, Mann–Whitney *U*-test was used. Spearman correlations-test with Bonferroni correction for multiple testing was used to analyze correlations between CPI and psychological distress as well as oral behaviors for the genotypes. As there were in total six comparisons for each genotype, Bonferroni correction was made to compensate for that, giving a significance level of *P* < 0.008.

Secondly, to explore if there were any interactions between *SERT* and the other genotypes with CPI, psychosocial distress, JFLS, and OBC linear models with interaction terms were used. Since these tests were pure exploratory analyses to generate new hypotheses, Bonferroni correction was not made.

Finally, we performed a factor analysis to describe the variability among the outcome variables, i.e., to determine if any unobserved factors were correlated to the outcome variables. These two latter analyses were carried out by a statistician using R 4.0.3 [50].

## Results

Background data of the participants are presented in Table 1. Most of the participants were women 20–40 years old, born in Scandinavia with a university degree. Most considered themselves healthy, but 56% reported at least one medical conditions. The most frequent were allergies (40%), ear-nose-throat disease (16%), and mental illness (10%). The participants used on average 0.7 (1.0) medications mostly analgesics. Almost 90% had a pain duration longer than 2 years with an average of 7.5 years. The pain was of on average of moderate intensity. Fourteen of the patients used antidepressant medicine, mostly serotonin reuptake inhibitors.

**Table 1:** Demographic data of 117 patients with TMD myalgia (16 men and 101 women).

**Demographic data**		**Frequency (*n*)**
Age (years)	Mean (SD)	38.2 (13.9)
	20–40	83
	>40	34
Sex	Male	16
	Female	101
Country of birth	Scandinavia	59
	Other European countries	11
	Africa	2
	Asia	40
	South America	1
	United states of America	1
	Missing data	3
Marital status	Married or living together	59
	Single	52
	Divorced or separated	2
	Missing data	4
Education	Elementary school	6
	High school	48
	University	59
	Missing data	4
Medical conditions	No medical condition	50
	=1	43
	>1	20
	Missing data	4
Number of medications	Mean (SD)	0.7 (1.0)
Pain duration (months)	Mean (SD)	90.3 (116.5)
	<6	0
	6–24	30
	>24	89
	Missing data	4
Current pain (NRS)	Mean (SD)	4.5 (2.3)
Characteristic pain (CPI)	Mean (SD)	53.8 (19.6)

As per the inclusion criteria, all patients were diagnosed with DC/TMD myalgia. Only 32% of the participants reported normal stress levels, while 45% reported moderate and 23% severe stress (PSS-10). Most reported normal anxiety scores, but 32% had mild, 10% moderate, and 4% severe anxiety (GAD-7). Furthermore, 35% reported mild, 13% moderate, and 8% severe depression (PHQ-9), while 36% reported low, 27% medium, and 13% high somatic symptoms (PHQ-15).

The frequencies of the genotypes are shown in Table 2. Some samples were undetermined in each SNP during analysis, hence reported as missing data. The most frequent genotype in the *HTR2A (rs9316233)* was homozygous common (C/C) followed by the heterozygote (C/G), while few individuals had the homozygous rare genotype (G/G). Similar results were found for the *HTR3A (rs1062613)*, where the homozygous common genotype (C/C) was most frequent followed by the heterozygote (C/T) and the homozygous rare (T/T) and the *HTR3B (rs1176744)*, where the homozygous common genotype (A/A) was most frequent, followed by the heterozygote (A/C) and the homozygous rare (C/C). In the *SERT (5-HTTLPR)* the most frequent genotype was the heterozygote (L/S), followed by the homozygous common (L/L) and lastly the homozygous rare (S/S). Also in the *COMT (rs4680)* the most common genotype was the heterozygote (A/G), followed by the homozygote rare (A/A), and the homozygous common (G/G). There as a significant difference between men and women in the frequency of genotypes for the *HTR3B (rs1176744) SNP* (Table 2).

**Table 2:** The distribution of *HTR2A (rs9316233), HTR3A (rs1062613), HTR3B (rs1176744), SERT (5-HTTLPR)* and *COMT (rs4680)* genotypes in 117 patients with TMD myalgia (16 men and 101 women).

***HTR2A*** ***n*** **=** **109**			***HTR3A*** ***n*** **=** **110**			***HTR3B*** ***n*** **=** **114**			***SERT*** ***n*** **=** **116**			***COMT*** ***n*** **=** **114**		
**C/C**	**C/G**	**G/G**	**C/C**	**C/T**	**T/T**	**A/A**	**A/C**	**C/C**	**S/S**	**L/S**	**L/L**	**A/A**	**A/G**	**G/G**
67	34	8	62	42	6	57	41	16	28	56	32	30	52	32

*The total number in each SNP varies since the genotypes could not be determined in some samples. Figures denote number of individuals*.

*HTR2A; HTR3A; HTR3B, serotonin type 2A, 3A, and 3B receptor gene, respectively. SERT, the serotonin transporter gene. COMT, catechol-O-methyltransferase gene. The frequencies of the genotypes differed significantly between men and women for the HTR3B SNP (P = 0.013), but there were no significant differences between women and men in frequencies for the other genotypes*.

**Figure 1:**
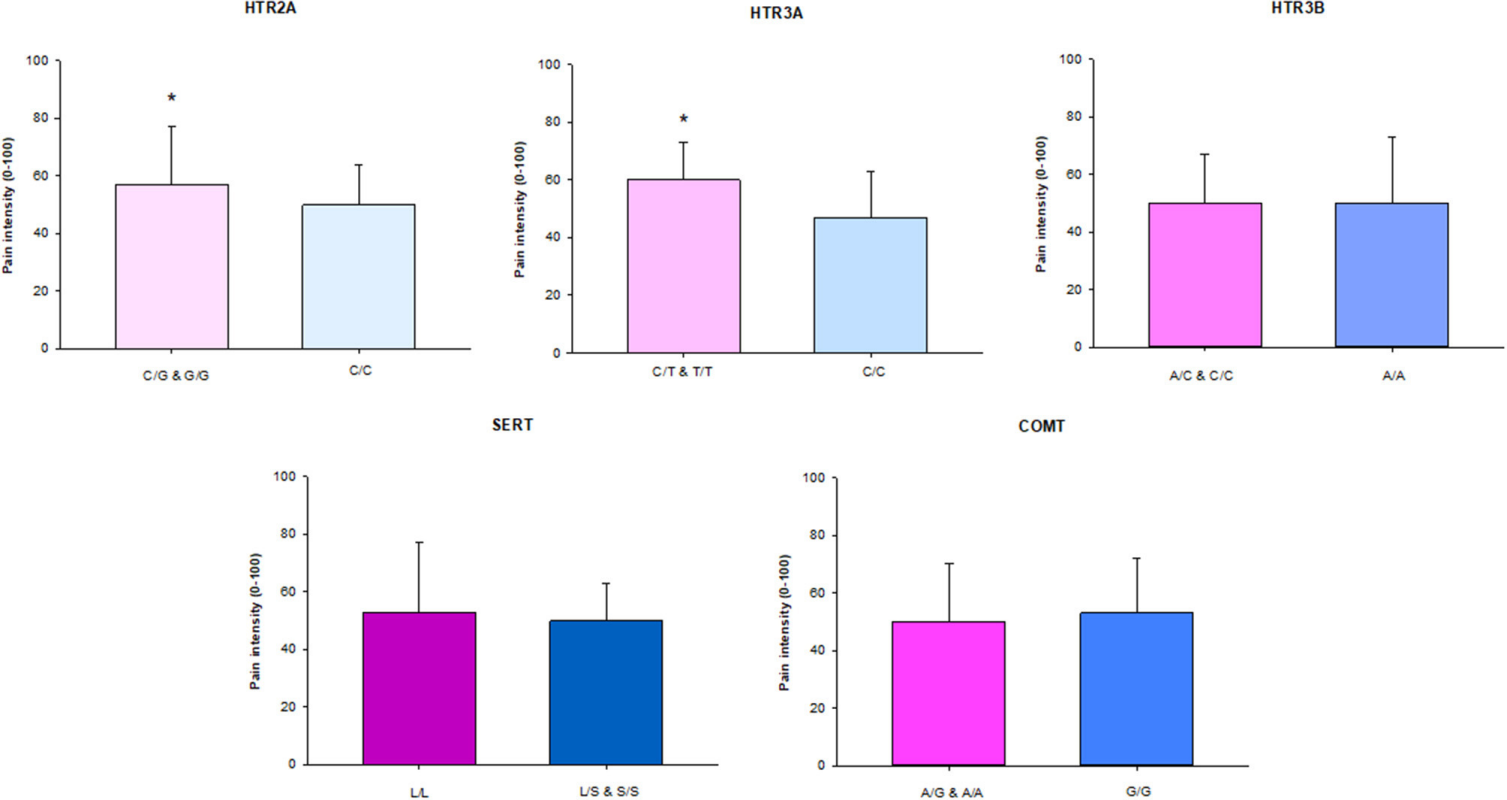
The median (IQR) characteristic pain intensity (CPI) of the SNPs in *HTR2A (rs9316233), HTR3A (rs1062613), HTR3B (rs1176744), SERT (5-HTTLPR*), and *COMT (rs4680)* in 117 individuals with TMD myalgia (16 men and 101 women). A, adenine; C, cytosine; G, guanine; T, thymine; S, short allele; L, long allele. There was a significant difference (*) in *HTR2A* and *HTR3A* SNPs with significant higher pain intensity in patients with the homozygous rare genotype in combination with heterozygote compared to the homozygous common genotype (*P* < 0.05).

Participants with the homozygous rare and heterozygous genotype in the *HTR2A* and *HTR3A* genes had significantly higher CPI than participants with the homozygous common genotypes (*P* = 0.042 and *P* = 0.024, respectively). There were no differences in the other genes (Figure 1).

Patients with the homozygous common genotype reported lower jaw functional limitation than patients with the other genotypes in the *HTR3A (rs1062613)* (*P* = 0.045). There were no other significant differences in psychosocial factors, jaw functional limitation, and oral behaviors between participants with different genotypes in the various genes (Table 3). Since 14 of the participants used antidepressants and this potentially could influence the results regarding psychological distress, we also ran analyses with these participants excluded. This did not change the results apart that a significant difference was observed between genotypes in the *COMT (rs4680)* with higher PHQ-15 values in the homozygous common genotype (*P* = 0.008).

**Table 3:** The median (IQR) scores for stress (PSS-10), anxiety (GAD-7), depression (PHQ-9), somatic symptoms (PHQ-15), jaw functional limitation (JFLS), and parafunctions (OBC) in the common homozygous and rare homozygous combined with the heterozygous genotypes of the *HTR2A (rs9316233), HTR3A (rs1062613), HTR3B (rs1176744), SERT (5-HTTLPR*), and *COMT (rs4680)* polymorphisms in 117 individuals with temporomandibular disorder myalgia.

	***HTR2A***		***HTR3A***		***HTR3B***		***SERT***		***COMT***	
	**C/C**	**C/G-G/G**	**C/C**	**C/T-T/T**	**A/A**	**A/C-C/C**	**L/L**	**L/S** **S/S**	**G/G**	**A/G** **A/A**
	***N* = 66**	***N* = 42**	***N* = 63**	***N* = 47**	***N* = 57**	***N* = 57**	***N* = 32**	***N* = 84**	***N* = 32**	***N* = 82**
PSS-10	16 (8)	16 (9)	16 (7)	16 (13)	16 (10)	16 (8)	16 (8)	14 (8)	17 (7)	15 (10)
GAD-7	4 (6)	5 (6)	4 (6)	4 (5)	4 (7)	4 (5)	5 (6)	4 (5)	5 (5)	4 (6)
PHQ-9	5 (7)	5 (6)	5 (7)	5 (6)	5 (7)	5 (8)	6 (8)	5 (2)	5 (7)	5 (6)
PHQ-15	8 (6)	11 (9)	8 (7)	9 (10)	7 (8)	9 (7)	12 (10)	8 (6)	11 (8)	8 (8)
JFLS	14 (36)	19 (24)	13 (32)	28 (37)	12 (32)	19 (40)	18 (36)	15 (36)	16 (39)	17 (35)
OBC	30 (10)	32 (19)	30 (10)	34 (16)	29 (11)	31 (15)	27 (14)	31 (33)	29 (10)	31 (14)

*HTR2A; HTR3A; HTR3B, serotonin type 2A, 3A, and 3B receptor gene; respectively. SERT, the serotonin transporter gene. COMT, catechol-O-methyltransferase gene. PSS, perceived stress scale; GAD, general anxiety disorder; PHQ, patient history questionnaire; JFLS, jaw functional limitation scale; OBC, oral behavior checklist. There were no significant differences between genotypes*.

We observed no deviation from Hardy–Weinberg equilibrium for any of the markers (*P* > 0.05).

The univariate correlation analyses showed several positive correlations between CPI on one hand, and self-reported psychosocial distress and jaw function on the other hand for several genotypes after correction for multiple testing (Table 4).

**Table 4:** Spearman's correlations coefficients (*r*_s_) between characteristic pain intensity on one hand, and psychological distress (PSS-10, GAD-7, PHQ-9, PHQ-15) and parafunctions (OBC) on the other hand, in 117 TMD myalgia patients grouped according to the common homozygous and rare homozygous combined with the heterozygous genotypes for the *HTR2A (rs9316233), HTR3A (rs1062613), HTR3B (rs1176744), SERT (5-HTTLPR)*, and *COMT (rs4680)* polymorphisms.

	***HTR2A***		***HTR3A***		***HTR3B***		***SERT***		***COMT***	
	**C/C**	**C/G G/G**	**C/C**	**C/T T/T**	**A/A**	**A/C C/C**	**L/L**	**L/S S/S**	**G/G**	**A/G A/A**
PSS-10	0.15	0.27	0.33	0.25	0.27	0.23	0.22	0.21	0.25	0.26
GAD-7	**0.37**	0.35	**0.42**	**0.41**	**0.49**	0.22	**0.28**	0.32	0.31	**0.40**
PHQ-9	0.23	**0.51**	**0.37**	**0.45**	**0.40**	0.27	**0.36**	0.33	0.37	**0.31**
PHQ-15	0.28	**0.47**	**0.46**	0.31	0.33	**0.41**	0.26	0.28	**0.53**	**0.33**
OBC	0.11	0.24	0.14	0.10	0.23	0.09	0.17	0.19	0.39	0.12
JFLS	0.26	**0.50**	**0.38**	**0.43**	**0.45**	0.30	**0.50**	0.33	**0.63**	0.29

*Characteristic pain intensity: The mean of the current as well as the average and worst pain intensity during the last month multiplicated with 10 (0–100). HTR2A; HTR3A; HTR3B, serotonin type 2A, 3A, and 3B receptor gene, respectively. SERT, the serotonin transporter gene. COMT, catechol-O-methyltransferase gene. PSS, perceived stress scale; GAD, generalized anxiety disorder; PHQ, patient health questionnaires; OBC, oral behaviors checklist; JFLS, jaw functional limitation scale. Bold figures denote significant correlations (P < 0.010 after Bonferroni correction)*.

In the exploratory analyses we found a few significant gene-to-gene interactions between *SERT* S/S and *SERT* L/S and other gene variants that correlated to psychological distress and jaw function, but also to pain (Table 5).

**Table 5:** Significant interactions between *SERT* rare homozygous (S/S) and heterozygous (L/S) genotypes and other genotypes on pain, psychological distress, and jaw function in 117 patients with TMD myalgia.

	**SERT S/S**	**SERT L/S**
HTR2A C/G	< JFLS (*P* = 0.008)	< CPI (*P* = 0.039)
HTR2A G/G		>PHQ-15 (*P* = 0.011)
HTR3A C/T		>JFLS (*P* = 0.027)
HTR3B C/C		>OBC (0.049)
COMT A/G	>PHQ-9 (*P* = 0.044)	
	>PSS-10 (*P* = 0.018)	
COMT G/G	>GAD-7 (*P* = 0.022)	>GAD-7 (*P* = 0.039)
	>PSS-10 (*P* = 0.041)	

*HTR2A; HTR3A; HTR3B, serotonin type 2A, 3A, and 3B receptor gene, respectively. SERT, the serotonin transporter gene. COMT, catechol-O-methyltransferase gene. PSS, perceived stress scale; GAD, generalized anxiety disorder; PHQ, patient health questionnaires; OBC, oral behaviors checklist; JFLS, jaw functional limitation scale*.

**Figure 2:**
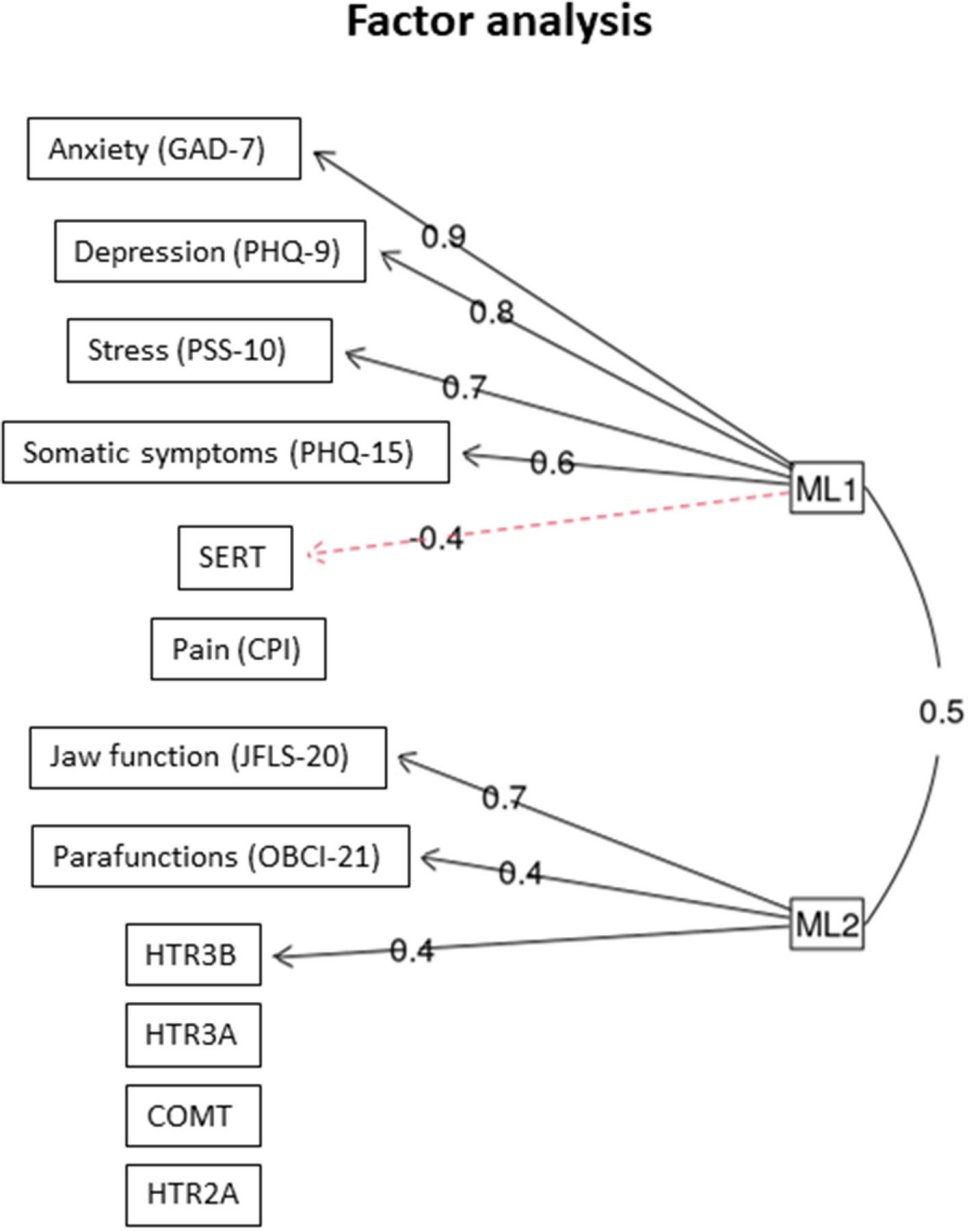
Factor analysis. The two latent factors that were identified (ML1 and ML 2) and the outcome variables they co-variated with *n* = 117 individuals with TMD myalgia. The figures denote factor loading. GAD, general anxiety disorder; PHQ, patient history questionnaire; PSS, perceived stress scale; CPI, characteristic pain intensity; JFLS, jaw functional limitation scale; OBC, oral behavior checklist.

In the factor analysis, two latent (unobserved) factors were found: The first factor (ML1) was positively associated with GAD-7, PHQ-9, PSS-10, and PHQ-15, and negatively correlated with *SERT*. The second factor (ML2) was positively associated to jaw function, self-reported parafunctions, and *HTR3B* (Figure 2).

## Discussion

The main results of this study was that TMD myalgia patients with the combined homozygous rare and heterozygous genotypes of the *HTR2A* (*rs9316233)* and *HTR3A (rs1062613) SNPs* had higher pain intensity (CPI) and jaw functional limitation (*HTR3A*) than patients with the common genotype. We also found positive correlations between pain intensity and psychological factors as well as self-reported oral behaviors for some of the genotypes. Additional exploratory analyses showed a few interactions between *SERT (5-HTTLPR)* and SNPs in the other genes, mainly to psychosocial distress and jaw function. Finally, factor analysis revealed two latent factors, one that was positively intercorrelated with the *HTR3B* gene, jaw function, and self-reported oral behaviors and another factor that was positively correlated with psychosocial distress and negatively to *SERT*. Hence, these findings strengthen the suggestion that polymorphisms in the serotonergic system may increase the vulnerability to both pain and psychosocial distress in chronic TMD.

The significant differences in pain intensity between carriers of the homozygous common and the combined group of the heterozygous and homozygous rare genotypes of the *HTR2A* (*rs9316233)* and *HTR3A (rs1062613)* indicates that the SNPs, substituting C to G and T, respectively, make a person more vulnerable to chronic pain. The difference in pain intensity was small for the *HTR2A* SNP but greater for *HTR3A*. Nevertheless, the pain experience is a complex phenomenon and influenced by many factors, for example previous pain experiences and emotional factors, that probably are more important. Out results regarding *HTR2A* (*rs9316233)* are in line with findings from the OPPERA (Orofacial Pain Prospective Evaluation and Risk Assessment) study [10]. However, that the *HTR3* receptor genes also seem to influence pain sensitivity in TMD is a novel finding. Regarding the other SNPs a few previous studies have reported an association between chronic TMD and genetic variations in *SERT* and *COMT* genes as well [10, 11, 51, 52], but these findings could not be replicated in our study.

Even if we found only a few differences in pain intensity between patients with the different serotonergic genotypes, there were several significant associations between pain and psychosocial distress for different SNPs which supports their close relationship and common pathways [31]. However, the correlations were in general weak to moderate, why other factors probably better explain this relationship. The *HTR3A (rs1062613)* polymorphism and its impact on emotional networks and depressed mood has earlier been investigated. One study showed that individuals with the homozygous common genotype had greater loss of gray matter in hippocampal structures compared to homozygous rare carriers suggesting that the common genotype may be associated with alterations in brain structures important for emotional processing, particularly when exposed to stress [53]. In this study pain intensity correlated significantly to anxiety, depression, and somatic symptoms in individuals with the *HTR3A (rs1062613)* homozygous common genotype. Furthermore, our findings showed that also in carriers of the common homozygous genotype in the *HTR3B (rs1176744)* SNP, pain intensity, and psychosocial distress correlated. The importance of the common genotype in psychiatric disorders is supported by a previous study showing that two distinct haplotypes of the A-allele in the *HTR3B (rs1176744)* SNP was associated with major depression [30]. In another study, the same *HTR3B* polymorphism was found to be associated with pain catastrophizing scores in healthy subjects [32]. However, for both the *HTR3A (rs1062613)* and *HTR3B (rs1176744)* there were significant correlations in individuals carrying at least one copy of the rare allele as well and for the *HTR2A* (*rs9316233)* there were instead stronger correlations between pain intensity and psychosocial distress in carriers of the rare allele. Therefore, these results must be interpreted with caution and probably more reflect associations that are independent on the serotonergic genotype.

It is well-known that anxiety traits are strongly linked to depression and anxiety disorders [54] and the literature supports the hypothesis that the *SERT 5-HTTLPR* polymorphism is associated with anxiety-related personality traits although there are inconsistent results [55]. The factor analysis in this study supports this by the identified latent factor associated with *SERT* and psychological distress. Several studies suggest that the homozygous rare genotype could be a risk factor for anxiety and major depression [55], posttraumatic stress disorder [56], and fibromyalgia [57]. However, even if most studies suggest the S-allele as a risk factor for psychosocial distress, other studies have suggested that the homozygous common genotype may also be a risk factor for psychological related personality traits [58, 59], in line with our results.

The *COMT* gene and its association with chronic pain and psychological traits has been a subject for many studies. Results suggest that the A-allele (rare genotype) in the SNP *(rs 4680)* is the risk allele for pain and pain sensitivity in chronic pain patients [60]. In this study we found no such difference between the genotypes on pain variables and psychosocial factors. However, we found correlations between pain and psychosocial distress especially in carriers of the A-allele. This contrasts another study showing that the common genotype was associated with depression in children exposed to a natural disaster [61]. The analyses of gene-to-gene interactions indicated that individuals with the *SERT (5-HTTLPR)* homozygous rare genotype in combination with one or two copies of the *COMT (rs4680)* G-allele seem to be more vulnerable to psychosocial distress.

Results from this study also showed that patients with the homozygous common genotype in the *HTR3B (rs1176744)* scored higher on jaw functional limitation and that jaw function, self-reported oral behaviors, and *HTR3B* co-variated with a latent factor. An association between TMD pain and self-reported oral behaviors and stress is in concordance to previously reports [62–64]. However, the association to the *HTR3B* gene is a new and interesting finding. We have previously shown a high expression of the 5-HT_3_ receptor in association to myocytes which may indicate that 5-HT have a role in motor function [65]. Hypothetically, the greater influence on oral behaviors and jaw function in homozygous common genotype of the *HTR3B (rs1176744)* in combination with *SERT (5-HTTLPR)* heterozygous genotype and *HTR3A (rs1062613)* heterozygous genotype in combination with the *SERT (5-HTTLPR)* heterozygous genotype could support this suggestion.

This study has a number of limitations that need to be addressed. One limitation is the uneven sized groups in each genotype. As mentioned above, very few individuals carried the *HTR2A* and the *HTR3A* rare homozygous genotypes why definite conclusions regarding their influence on pain and psychosocial distress cannot be drawn. It can therefore be argued that the study was underpowered to detect any significant differences. To solve this issue the homozygous rare genotypes were combined with the heterozygous genotype in the analyses. As a result, most groups were of similar size and exceeded the 37 participants per genotype that we had calculated as minimum to achieve statistical significance with sufficient power. Nevertheless, to prevent this in future studies, a larger population or groups of gene variants with similar sizes [32], would be preferable. Further, since a proportion of participants reported mental illness the relation between pain and psychological distress may have been overestimated. Similar, 14 patients took antidepressant medicine which also could influence the results. Excluding these participants only changed the results to a minor extent which is why we decided to include them. However, this could be taken into consideration in future studies. Another limitation is that few men were included so we did not consider it relevant to analyze sex differences. Because of this limitation the results cannot fully be extrapolated to men. However, it is well-known that there are more women with TMD disorders; hence, our patient sample reflects the TMD population [4]. Also, in future studies it will be beneficial to simultaneously consider additional polymorphisms, both independently and combined, as well as environmental factors in order to learn more about the pathophysiology of chronic pain disorders and psychosocial factors. Obviously, genes do not operate independently, but function against a background of other essential factors. Also the interplay with a wide and multifaceted range of other risk factors over time would need to be taken into account to better understand initiation and maintenance of TMD pain [9].

Taken together and considering the limitations of this study, we conclude that the polymorphisms *rs9316233* and *rs1062613* in the *HTR2A* and *HTR3A* genes, respectively, contribute to pain intensity in TMD myalgia. This together with interactions between pain variables and psychosocial factors in several of the genes investigated strengthens that pain and psychosocial distress are, indeed, related. Additional analyses revealed a latent factor that interacted with the *HTR3B* gene, jaw function and self-reported oral behaviors, which may implicate a role for the 5-HT_3_ receptor for jaw motor function. Further research is needed to explore this as well as the influence of gene-to-gene interactions on pain and psychosocial distress.

## Data Availability Statement

The raw data supporting the conclusions of this article will be made available by the authors on request, without undue reservation.

## Ethics Statement

The study was reviewed and approved by The Regional Ethical Review Board in Stockholm, Sweden (2011/1955-31/2) and the Swedish Data Protection Authority (Dnr 54-2013). The patients/participants provided their written informed consent to participate in this study.

## Author Contributions

NC, TL, MS, and ME: conceptualization. SL, NC, MS, and ME: methodology. SL, NC, and ME: software, validation, formal analysis, and data curation. SL and NC: investigation. SL, NC, BH-M, MS, and ME: resources. SL: writing (original draft preparation) and visualization. SL, NC, BH-M, TL, PS, MS, and ME: writing (review and editing). NC, PS, and ME: supervision. NC and ME: project administration. BH-M and ME: funding acquisition. All authors contributed to the article and approved the submitted version.

## Conflict of Interest

The authors declare that the research was conducted in the absence of any commercial or financial relationships that could be construed as a potential conflict of interest.
